# Assessment of early rib hump deformity correction in adolescent idiopathic scoliosis treated with a dynamic derotation brace using the double rib contour sign

**DOI:** 10.1186/1748-7161-8-S2-O54

**Published:** 2013-09-18

**Authors:** Theodoros Grivas, Georgios Triantafyllopoulos, Christina Mazioti

**Affiliations:** 1 Scoliosis Clinic of the Department of Trauma and Orthopaedics, "Tzaneio" General Hospital, Piraeus, Greece

## Background

Scoliotic children and their parents are very much concerned about trunk deformity (TD). One of the TD components is the rib hump (RH), which is mainly the expression of rib deformity. Bracing treatment aims not only to hold or correct the central axis (i.e. the spine), but also the TD in the thorax (i.e., the RH). 

## Purpose

The goal of this study was to assess the initial correction of the RH in patients with AIS who were treated with the Dynamic Derotation Brace (DDB). 

## Methods

In total, 20 children with right thoracic (n= 14) and double curves (n=6) (right thoracic left lumbar) were assessed. The SRS/SOSORT inclusion criteria for brace treatment were used. The Cobb angle was measured on postero-anterior and the rib index (RI) was calculated from the double rib contour sign (DRCS) according to Grivas et al. 2002 on lateral standing spinal radiographs. The reference vertebra from which the RI was assessed was documented. Statistical analysis was done using the Statistical Package Social Science (SPSS) using the t-test. 

## Results

The mean thoracic Cobb angle was 27.5 degrees. The posterior margin of the reference vertebra was the T8 in four scoliotics, T9 in two, T10 in four, T11 in six, L1 in two and L2 in two, respectively. The mean pre-brace treatment RI was 1,864 and the early post-brace 1,205, respectively, p=0,007. 

## Conclusions and discussion

The RI resulting from the DRCS for the first time was used to assess RH deformity in scoliotic children during brace treatment. The RI was used due to its simplicity and the ability to be calculated on the lateral scoliosis film with no need for special imaging or additional exposure to radiation. The DDB significantly improved the RH deformity during the initial treatment period in the thoracic curves and in the thoracic component of the double scoliotic curves. The impressive improvement of the RI can be attributed to the metallic blades featuring DDB. The RI based on DRCS could easily be used to assess any brace effectiveness on the RH deformity correction. 
